# Supplementary material for: Com1 as a Promising Protein for the Differential Diagnosis of the Two Forms of Q Fever
Source: Pathogens. 2019 Nov 18;8(4):242. doi: 10.3390/pathogens8040242 (PMC6963606; doi:10.3390/pathogens8040242)
Supplement: Supplementary file 1 [file pathogens-08-00242-s001.pdf]

1 tggcgaatgg gacgcgccct gtagcggcgc attaagcgcg gcgggtgtgg tggttacgcg

61 cagcgtgacc gctacacttg ccagcgccct agcgcccgct cctttcgctt tcttcccttc

121 ctttctcgcc acgttcgccg gctttccccg tcaagctcta aatcgggggc tccctttagg

181 gttccgattt agtgctttac ggcacctcga ccccaaaaaa cttgattagg gtgatggttc

241 acgtagtggg ccatcgccct gatagacggt ttttcgccct ttgacgttgg agtccacgtt

301 ctttaatagt ggactcttgt tccaaactgg aacaacactc aaccctatct cggtctattc

361 ttttgattta taagggattt tgccgatttc ggcctattgg ttaaaaaatg agctgattta

421 acaaaaattt aacgcgaatt ttaacaaaat attaacgttt acaatttcag gtggcacttt

481 tcggggaaat gtgcgcggaa cccctatttg tttatttttc taaatacatt caaatatgta

541 tccgctcatg agacaataac cctgataaat gcttcaataa tattgaaaaa ggaagagtat

601 gagtattcaa catttccgtg tcgcccttat tccctttttt gcggcatttt gccttcctgt

661 ttttgctcac ccagaaacgc tggtgaaagt aaaagatgct gaagatcagt tgggtgcacg

721 agtgggttac atcgaactgg atctcaacag cggtaagatc cttgagagtt ttcgccccga

781 agaacgtttt ccaatgatga gcacttttaa agttctgcta tgtggcgcgg tattatcccg

841 tattgacgcc gggcaagagc aactcggtcg ccgcatacac tattctcaga atgacttggt

901 tgagtactca ccagtcacag aaaagcatct tacggatggc atgacagtaa gagaattatg

961 cagtgctgcc ataaccatga gtgataacac tgcggccaac ttacttctga caacgatcgg

1021 aggaccgaag gagctaaccg cttttttgca caacatgggg gatcatgtaa ctcgccttga

1081 tcgttgggaa ccggagctga atgaagccat accaaacgac gagcgtgaca ccacgatgcc

1141 tgcagcaatg gcaacaacgt tgcgcaaact attaactggc gaactactta ctctagcttc

1201 ccggcaacaa ttaatagact ggatggaggc ggataaagtt gcaggaccac ttctgcgctc

1261 ggcccttccg gctggctggt ttattgctga taaatctgga gccggtgagc gtgggtctcg

1321 cggtatcatt gcagcactgg ggccagatgg taagccctcc cgtatcgtag ttatctacac

1381 gacggggagt caggcaacta tggatgaacg aaatagacag atcgctgaga taggtgcctc

1441 actgattaag cattggtaac tgtcagacca agtttactca tatatacttt agattgattt

1501 aaaacttcat ttttaattta aaaggatcta ggtgaagatc ctttttgata atctcatgac

1561 caaaatccct taacgtgagt tttcgttcca ctgagcgtca gaccccgtag aaaagatcaa

1621 aggatcttct tgagatcctt tttttctgcg cgtaatctgc tgcttgcaaa caaaaaaacc

1681 accgctacca gcggtggttt gtttgccgga tcaagagcta ccaactcttt ttccgaaggt

1741 aactggcttc agcagagcgc agataccaaa tactgtcctt ctagtgtagc cgtagttagg

1801 ccaccacttc aagaactctg tagcaccgcc tacatacctc gctctgctaa tcctgttacc

1861 agtggctgct gccagtggcg ataagtcgtg tcttaccggg ttggactcaa gacgatagtt

1921 accggataag gcgcagcggt cgggctgaac ggggggttcg tgcacacagc ccagcttgga

1981 gcgaacgacc tacaccgaac tgagatacct acagcgtgag ctatgagaaa gcgccacgct

2041 tcccgaaggg agaaaggcgg acaggtatcc ggtaagcggc agggtcggaa caggagagcg

2101 cacgagggag cttccagggg gaaacgcctg gtatctttat agtcctgtcg ggtttcgcca

2161 cctctgactt gagcgtcgat ttttgtgatg ctcgtcaggg gggcggagcc tatggaaaaa

2221 cgccagcaac gcggcctttt tacggttcct ggccttttgc tggccttttg ctcacatgtt

2281 ctttcctgcg ttatcccctg attctgtgga taaccgtatt accgcctttg agtgagctga

2341 taccgctcgc cgcagccgaa cgaccgagcg cagcgagtca gtgagcgagg aagcggaaga

2401 gcgcctgatg cggtattttc tccttacgca tctgtgcggt atttcacacc gcatatatgg

2461 tgcactctca gtacaatctg ctctgatgcc gcatagttaa gccagtatac actccgctat

2521 cgctacgtga ctgggtcatg gctgcgcccc gacacccgcc aacacccgct gacgcgccct

2581 gacgggcttg tctgctcccg gcatccgctt acagacaagc tgtgaccgtc tccgggagct

2641 gcatgtgtca gaggttttca ccgtcatcac cgaaacgcgc gaggcagctg cggtaaagct

2701 catcagcgtg gtcgtgaagc gattcacaga tgtctgcctg ttcatccgcg tccagctcgt

2761 tgagtttctc cagaagcgtt aatgtctggc ttctgataaa gcgggccatg ttaagggcgg

2821 tttttcctg tttggtcact gatgcctccg tgtaaggggg atttctgttc atgggggtaa

2881 tgataccgat gaaacgagag aggatgctca cgatacgggt tactgatgat gaacatgccc

2941 ggttactgga acgttgtgag ggtaaacaac tggcggtatg gatgcggcgg gaccagagaa

3001 aaatcactca gggtcaatgc cagcgcttcg ttaatacaga tgtaggtgtt ccacagggta

3061 gccagcagca tcctgcgatg cagatccgga acataatggt gcagggcgct gacttccgcg

3121 tttccagact ttacgaaaca cggaaaccga agaccattca tgttgttgct caggtcgcag

3181 acgttttgca gcagcagtcg cttcacgttc gctcgcgtat cggtgattca ttctgctaac

3241 cagtaaggca accccgccag cctagccggg tcctcaacga caggagcacg atcatgcgca

3301 cccgtggggc cgccatgccg gcgataatgg cctgcttctc gccgaaacgt ttggtggcgg

3361 gaccagtgac gaaggcttga gcgagggcgt gcaagattcc gaataccgca agcgacaggc

3421 cgatcatcgt cgcgctccag cgaaagcggt cctcgccgaa aatgacccag agcgctgccg

3481 gcacctgtcc tacgagttgc atgataaaga agacagtcat aagtgcggcg acgatagtca

3541 tgccccgcgc ccaccggaag gagctgactg ggttgaaggc tctcaagggc atcggtcgag

3601 atcccggtgc ctaatgagtg agctaactta cattaattgc gttgcgctca ctgcccgctt

3661 tccagtcggg aaacctgtcg tgccagctgc attaatgaat cggccaacgc gcggggagag

3721 gcggtttgcg tattgggcgc cagggtggtt tttcttttca ccagtgagac gggcaacagc

3781 tgattgccct tcaccgcctg gccctgagag agttgcagca agcggtccac gctggtttgc

3841 cccagcaggc gaaaatcctg tttgatggtg gttaacggcg ggatataaca tgagctgtct

3901 tcggtatcgt cgtatcccac taccgagata tccgcaccaa cgcgcagccc ggactcggta

3961 atggcgcgca ttgcgcccag cgccatctga tcgttggcaa ccagcatcgc agtgggaacg

4021 atgccctcat tcagcatttg catggtttgt tgaaaaccgg acatggcact ccagtcgcct

4081 tcccgttccg ctatcggctg aatttgattg cgagtgagat atttatgcca gccagccaga

4141 cgcagacgcg ccgagacaga acttaatggg cccgctaaca gcgcgatttg ctggtgaccc

4201 aatgcgacca gatgctccac gcccagtcgc gtaccgtctt catgggagaa aataatactg

4261 ttgatgggtg tctggtcaga gacatcaaga aataacgccg gaacattagt gcaggcagct

4321 tccacagcaa tggcatcctg gtcatccagc ggatagttaa tgatcagccc actgacgcgt

4381 tgcgcgagaa gattgtgcac cgccgcttta caggcttcga cgccgcttcg ttctaccatc

4441 gacaccacca cgctggcacc cagttgatcg gcgcgagatt taatcgccgc gacaatttgc

4501 gacggcgcgt gcagggccag actggaggtg gcaacgccaa tcagcaacga ctgtttgccc

4561 gccagttgtt gtgccacgcg gttgggaatg taattcagct ccgccatcgc cgcttccact

4621 ttttcccgcg ttttcgcaga aacgtggctg gcctggttca ccacgcggga aacggtctga

4681 taagagacac cggcatactc tgcgacatcg tataacgtta ctggtttcac attcaccacc

4741 ctgaattgac tctcttccgg gcgctatcat gccataccgc gaaaggtttt gcgccattcg

4801 atggtgtccg ggatctcgac gctctccctt atgcgactcc tgcattagga agcagcccag

4861 tagtaggttg aggccgttga gcaccgccgc cgcaaggaat ggtgcatgca aggagatggc

4921 gcccaacagt cccccggcca cggggcctgc caccataccc acgccgaaac aagcgctcat

4981 gagcccgaag tggcgagccc gatcttcccc atcggtgatg tcggcgatat aggcgccagc

5041 aaccgcacct gtggcgccgg tgatgccggc cacgatgcgt ccggcgtaga ggatcgagat

5101 ctcgatcccg cgaaattaat acgactcact ataggggaat tgtgagcgga taacaattcc

5161 cctctagaaa taattttgtt taactttaag aaggagatat acatatgaag aaccgtttga

5221 ctgcactatt tttagccgga accttgaccg caggcgtggc gatagccgcc ccctctcaat

5281 tcagtttttc tcctcaacaa gtcaaagaca tacaaagcat cgtccaccat tatttagtca

5341 accacccaga agttttagta gaagcatccc aagcattgca aaaaaagaca gaagcgcaac

5401 aagaagaaca cgctcaacaa gcaattaaag aaaatgcaaa gaaattattt aacgaccctg

5461 catcaccagt ggcaggcaat cctcatggca atgttacatt ggttgaattt ttcgattatc

5521 aatgtggcca ttgcaaagcc atgaattctg ttattcaagc tatcgtgaaa caaaataaaa

5581 acctccgcgt tgtcttcaaa gaactgccca tttttggcgg ccaatcgcaa tacgctgcca

5641 aagtatcatt agcagccgct aaacaaggaa aatattatgc tttccacgac gcgctgctca

5701 gtgtcgacgg ccaattatca gaacaaatca cccttcaaac cgcagaaaaa gtaggattaa

5761 atgttgctca gctcaaaaaa gacatggata atcctgctat ccaaaaacaa ctgcgtgata

5821 acttccaatt agctcaatcg ttacagctag caggcacccc gacgttcgtc attggtaata

5881 aagcgttaac caaattcggt tttatacccg gcgccacctc acaacaaaac cttcaaaaag

5941 aaatcgaccg ggtagaaaag ctcgagcacc accaccacca ccactgagat ccggctgcta

6001 acaaagcccg aaaggaagct gagttggctg ctgccaccgc tgagcaataa ctagcataac

6061 cccttggggc ctctaaacgg gtcttgaggg gttttttgct gaaaggagga actatatccg

6121 gat
